# Specific and Efficient Targeting of Cyanobacterial Bicarbonate Transporters to the Inner Envelope Membrane of Chloroplasts in *Arabidopsis*

**DOI:** 10.3389/fpls.2016.00016

**Published:** 2016-02-02

**Authors:** Susumu Uehara, Fumi Adachi, Yasuko Ito-Inaba, Takehito Inaba

**Affiliations:** ^1^Department of Agricultural and Environmental Sciences, Faculty of Agriculture, University of MiyazakiMiyazaki, Japan; ^2^Organization for Promotion of Tenure Track, University of MiyazakiMiyazaki, Japan

**Keywords:** bicarbonate transporter, chloroplast, *Arabidopsis*, protein targeting, cyanobacteria

## Abstract

Installation of cyanobacterial bicarbonate transporters to the inner envelope membrane (IEM) of chloroplasts in C_3_ plants has been thought to improve photosynthetic performance. However, the method to deliver cyanobacterial bicarbonate transporters to the chloroplast IEM remains to be established. In this study, we provide evidence that the cyanobacterial bicarbonate transporters, BicA and SbtA, can be specifically installed into the chloroplast IEM using the chloroplast IEM targeting signal in conjunction with the transit peptide. We fused the transit peptide and the mature portion of Cor413im1, whose targeting mechanism to the IEM has been characterized in detail, to either BicA or SbtA isolated from *Synechocystis* sp. PCC6803. Among the seven chimeric constructs tested, we confirmed that four chimeric bicarbonate transporters, designated as BicAI, BicAII, SbtAII, and SbtAIII, were expressed in *Arabidopsis*. Furthermore, these chimeric transporters were specifically targeted to the chloroplast IEM. They were also resistant to alkaline extraction but can be solubilized by Triton X-100, indicating that they are integral membrane proteins in the chloroplast IEM. One of the transporters, BicA, could reside in the chloroplast IEM even after removal of the IEM targeting signal. Taken together, our results indicate that the addition of IEM targeting signal, as well as the transit peptide, to bicarbonate transporters allows us to efficiently target nuclear-encoded chimeric bicarbonate transporters to the chloroplast IEM.

## Introduction

Ribulose 1,5-bisphosphate carboxylase/oxygenase (Rubisco) is the enzyme that catalyzes the incorporation of CO_2_ into ribulose 1, 5-bisphosphate (RuBP), resulting in the production of two 3-phosphoglycerate (3-PGA) molecules (Whitney et al., 2011). This reaction is known as the first step of inorganic carbon fixation of photosynthesis. In addition to the carboxylation reaction, Rubisco also catalyzes the oxygenation of RuBP. The oxygenation of RuBP produces one molecule of 2-phosphoglycerate (2-PG), as well as one molecule of 3-PGA. However, 2-PG cannot be utilized by the Calvin cycle and must be recycled back into 3-PGA via the photorespiration pathway. This recycling process results in a partial loss of CO_2_, and energy consumption in C_3_ plants, and has been thought to make photosynthesis in C_3_ plants inefficient (Price et al., 2013).

Cyanobacteria and algae have evolved unique CO_2_-concentrating mechanisms (CCMs) to overcome this problem (Price et al., 2008, 2013). In cyanobacterial CCM, the key components are CO_2_/bicarbonate transporters, and the microcompartments called carboxysomes, which contain Rubisco. At least five distinct inorganic carbon (Ci) transporters have been identified to date (Price et al., 2008, 2013). BicA and SbtA are single-subunit, Na^+^-dependent bicarbonate transporters at the plasma membrane (Shibata et al., 2002; Price et al., 2004). Both genes are strongly induced by inorganic carbon limitation, and disruption of either gene impairs the photosynthetic capacity in cyanobacteria (Shibata et al., 2002; Price et al., 2004). In contrast, BCT1 is an ATP-binding cassette type bicarbonate transporter, composed of multiple subunits, and thought to utilize ATP as the energy for HCO_3_^-^ transport (Omata et al., 1999). In addition to the HCO_3_^-^ transport systems, NDH-I type CO_2_ uptake systems are located at the thylakoid membranes and convert CO_2_ to HCO_3_^-^, thereby preventing the leakage of CO_2_ from the cell (Klughammer et al., 1999; Ohkawa et al., 2000).

It has been proposed that the installation of CCMs into chloroplasts is a promising approach to improve photosynthesis in C_3_ plants (Price et al., 2013; Price and Howitt, 2014a). According to a theoretical estimation, installation of BicA and SbtA into the chloroplast inner envelope membrane (IEM) improves photosynthetic CO_2_ fixation rates (Price et al., 2013). Hence, installation of functional BicA and SbtA into the chloroplast IEM is becoming one of prime targets for the improvement of photosynthesis (Price and Howitt, 2014a). A major technical challenge is how to deliver bicarbonate transporters specifically to the chloroplast IEM. In cyanobacteria, bicarbonate transporter proteins are thought to be inserted into the plasma membrane from the cytosol (Frain et al., 2015). In land plant chloroplasts, at least two chloroplast-encoded proteins, Ycf1/Tic214 and CemA, have been shown to be inserted into the IEM from the stroma (Sasaki et al., 1993; Kikuchi et al., 2013). This indicates that plastid-encoded membrane proteins can be targeted to the IEM. However, when the cyanobacterial bicarbonate transporter, BicA, was expressed from the plastid genome, the vast majority of the expressed BicA was targeted to the thylakoid membranes instead of the IEM (Pengelly et al., 2014). Because virtually nothing is known about the mechanism by which plastid-encoded membrane proteins are integrated into the chloroplast IEM, it remains a challenge to install plastid-encoded bicarbonate transporters precisely to the IEM.

As an alternative, the installation of nuclear-encoded bicarbonate transporters to the chloroplast IEM can be employed. It has been shown that IEM proteins utilize two distinct pathways for their targeting to the IEM (Inaba and Schnell, 2008; Oh and Hwang, 2015). One is the stop-transfer pathway and the other is the post-import or conservative pathway. To date, aside from Tic40 and Tic110, all the IEM proteins investigated seem to utilize the stop-transfer pathway, suggesting that a stop-transfer mechanism plays a key role in the biogenesis of IEM proteins (Lubeck et al., 1997; Li and Schnell, 2006; Tripp et al., 2007; Firlej-Kwoka et al., 2008; Viana et al., 2010; Okawa et al., 2014). Both stop-transfer and post-import substrate proteins have bipartite signals, that is, they are composed of the transit peptide and IEM targeting signal (Inaba and Schnell, 2008; Oh and Hwang, 2015). The transit peptide is predictable, usually located at the N-terminus of precursor proteins, and cleaved off after the import into chloroplasts. In contrast, the IEM targeting signal is usually retained within the mature portion (Inaba and Schnell, 2008; Oh and Hwang, 2015). Our previous study demonstrated that the IEM targeting signal is sufficient to deliver the chimeric protein to the chloroplast IEM *in vivo* (Okawa et al., 2014). This suggests that the addition of the IEM targeting signal, as well as the transit peptide to bicarbonate transporters, enables us to install nuclear-encoded bicarbonate transporters into the chloroplast IEM.

In this study, we examined the installation of nuclear-encoded cyanobacterial bicarbonate transporters, BicA and SbtA, to the IEM of chloroplasts in *Arabidopsis*. We successfully expressed chimeric BicA and SbtA proteins in *Arabidopsis* chloroplasts. Furthermore, both chimeric bicarbonate transporters specifically accumulated to the IEM. One of the transporters, BicA, could reside in the chloroplast IEM even after removal of the IEM targeting signal. Based on these results, we propose a new approach to targeting nuclear-encoded cyanobacterial bicarbonate transporters to the chloroplast IEM by using chimeric constructs.

## Materials and Methods

### Construction of Vectors and *Arabidopsis* Transformation

The *bicA* and *sbtA* genes were amplified from the genomic DNA of *Synechocystis* sp. PCC6803.

All the plasmids used to amplify each portion are described previously (Okawa et al., 2014). Primers used to amplify each portion are listed in Supplementary Figure S1. For the construction of BicAI and SbtAI, the TP-Cor413im1-protein A portion was amplified using pET/pre-Cor413im1-pA as the template (Okawa et al., 2014). For the construction of BicAII, BicAIII, SbtAII, and SbtAIII, the Cor413im1-Protein A and the K124-Protein A portions were amplified using pET/pre-Cor413im1-pA and pET/TP-K124-pA as the templates, respectively. Both the TP-protein A and Cor413im1 portions of BicAIV was amplified using pET/TP-pA-Cor413im1. The transit peptide portion of BicAII, BicAIII, SbtAII, and SbtAIII, was amplified using pET/TP-pA-Cor413im1.

**FIGURE 1:**
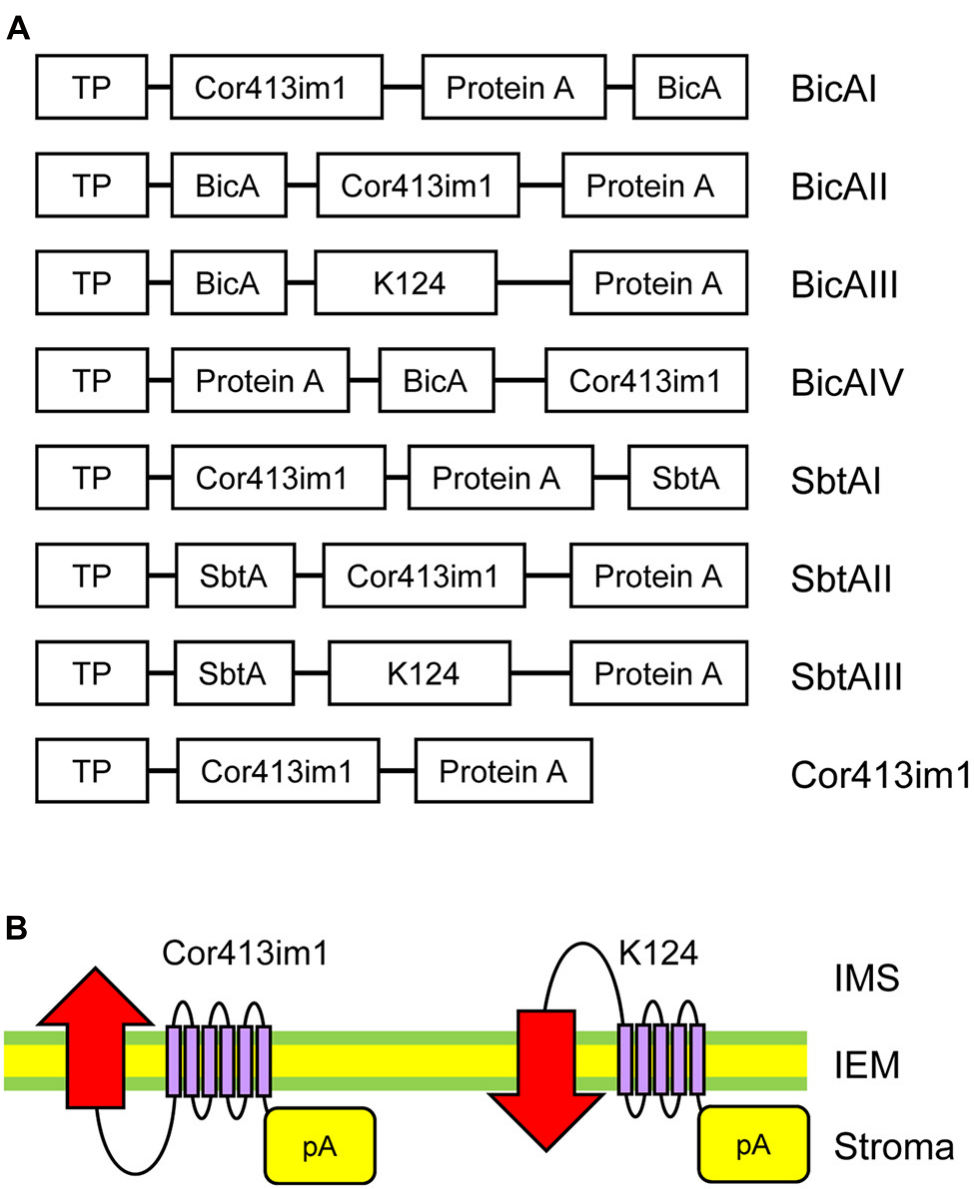
**Construct designs for specific targeting of bicarbonate transporters to the chloroplast IEM. (A)** Schematic diagram of chimeric BicA and SbtA constructs used in this study. The “protein A” domain of the fusion constructs contains two IgG-binding domains from staphylococcal protein A. Both *bicA* and *sbtA* genes are derived from *Synechocystis* sp. PCC 6803. The K124 construct lacks the sixth transmembrane domain of Cor413im1. TP, the transit peptide of Cor413im1. **(B)** Possible flipping of bicarbonate transporters at the chloroplast IEM by truncated Cor413im1. A previous study demonstrated that the protein A portion fused to the C-terminus of K124, and full length Cor413im1, is located in the stroma. Due to the lack of the sixth transmembrane segment, the N-terminus of K124 is predicted to be exposed to the intermembrane space (right). Hence, the transmembrane topology of the bicarbonate transporter (indicated by red arrows) fused to K124 is assumed to be flipped as compared to that fused to full-length Cor413im1 (left).

After amplification of all these fragments, multiple DNA fragments were simultaneously sub-cloned into either *Nco*I-*Xba*I sites of pCAMBIA3300 (for BicA), or *Nco*I-*Nhe*I sites of pCAMBIA1301 (for SbtA) using an In-Fusion HD Cloning Kit (Takara) to obtain the constructs summarized in **Figure 1**.

All pCAMBIA constructs were introduced into *Arabidopsis thaliana* (accession Columbia) via *Agrobacterium tumefaciens*-mediated transformation using the floral dip method (Clough and Bent, 1998).

### Plant Material and Growth Conditions

Wild type (WT, accession Columbia) and transgenic plants expressing chimeric BicA or SbtA proteins were grown at 22°C under continuous light conditions.

### *Arabidopsis* Chloroplast Isolation and Membrane Fractionation

For chloroplast isolation, *Arabidopsis* plants were grown on 0.5 × MS plates supplemented with 1% sucrose at 22°C. Chloroplasts were isolated from 14- to 18-day-old transgenic plants expressing BicA or SbtA, as described previously (Smith et al., 2002).

For the preparation of total chloroplast membrane and soluble proteins, isolated chloroplasts were diluted in either 0.2 M Na_2_CO_3_ (pH 12.0) or 1% Triton X-100, and incubated for 10 min on ice. The samples were then separated into soluble and membrane fractions by ultracentrifugation at 100,000 × *g* for 15 min.

### Analysis of the Localization of Truncated Proteins within Chloroplasts

To determine the localization of each chimeric protein within chloroplasts, isolated chloroplasts were fractionated into stroma, envelope, and thylakoid membranes as described previously (Smith et al., 2002). After the quantification of proteins in each fraction, total chloroplast (3 μg), stroma (3 μg), envelope (1 μg), and thylakoid (1.5 μg) fractions were analyzed by sodium dodecyl sulfate-polyacrylamide gel electrophoresis (SDS-PAGE), and immunoblotted with the antisera indicated in the figures. Although we sometimes loaded a different amount of proteins for the analysis, the protein ratio of total chloroplast:stroma:envelope:thylakoid was consistently 3:3:1:1.5. The trypsin sensitivity of chimeric BicA and SbtA proteins was examined using intact chloroplasts, as described previously (Jackson et al., 1998; Li and Schnell, 2006).

For the tobacco etch virus (TEV) protease treatment, inside-out envelope membrane vesicles were prepared as described previously (Li and Schnell, 2006; Okawa et al., 2008). The inside-out envelope membrane vesicles of BicAI chloroplasts were treated with TEV protease at 30°C for 1 h. After TEV protease treatment, the vesicles were diluted in 0.2 M Na_2_CO_3_ (pH 12.0), incubated for 10 min on ice, and then separated into soluble and membrane fractions by ultracentrifugation at 100,000 × *g* for 15 min.

Antibodies against LSU, Tic110, and Toc75 are described previously (Sasaki et al., 1981; Inaba et al., 2005; Okawa et al., 2014). The LHCP antibodies were a kind gift from Prof. Kenneth Cline. The anti-protein A IgG was purchased from Sigma–Aldrich.

The fold enrichment of each chimeric protein in the envelope fraction was estimated using densitometric software (CS analyzer, ATTO) as described previously (Okawa et al., 2014). As controls, we also estimated the fold enrichment of Tic110 and LHCP in the envelope fraction.

## Results

### Expression of Nuclear-Encoded Chimeric Bicarbonate Transporters in *Arabidopsis*

**FIGURE 2:**
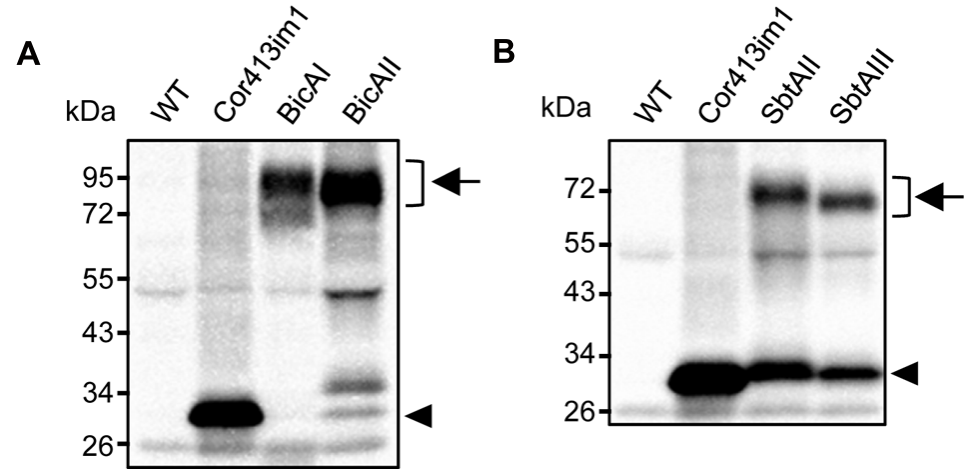
**Expression analysis of chimeric BicA **(A)** and SbtA **(B)** in transgenic *Arabidopsis*.** Total protein extracts (20 μg for Cor413im1, 40 μg for others) from true leaves were resolved by SDS-PAGE and probed with antibodies against protein A. Chimeric bicarbonate transporters are indicated by arrows. Arrowheads indicate the position of Cor413im1-protein A. Note that Cor413im1-protein A found in SbtAI and SbtAII are likely to be degradation products.

Installation of cyanobacterial bicarbonate transporters into the IEM of chloroplasts is challenging due to the lack of techniques allowing specific targeting of those transporters to the chloroplast IEM. To overcome this issue, we took advantage of the chimeric expression approach using a chloroplast IEM protein as a fusion partner. In our previous studies, we demonstrated that the Cold-regulated 413IM1 (Cor413IM1) protein is a chloroplast IEM protein, and indeed has the IEM targeting signal within the mature portion (Okawa et al., 2008, 2014). Hence, we generated the seven chimeric constructs shown in **Figure 1A**. In these constructs, the transit peptide and mature portion of Cor413im1, and *Staphylococcus* protein A, were fused to either BicA or SbtA, which are bicarbonate transporters found in cyanobacteria (**Figure 1A**). We also included K124 constructs lacking the sixth transmembrane domain of Cor413im1 as a fusion partner. According to our previous study (Okawa et al., 2014), the topology of K124 has been shown to be flipped at the IEM, and the C-terminus faces toward the stroma (**Figure 1B**). Hence, we assumed that the topology of a bicarbonate transporter fused to K124 is reverted, as compared to those fused to the full-length Cor413im1 (**Figure 1B**). All these constructs were transformed into *Arabidopsis* by *Agrobacterium*-mediated transformation. Among the seven chimeric bicarbonate transporter constructs we tested, we confirmed the expression of four chimeric proteins; BicAI, BicAII, SbtAII, and SbtAIII (**Figures 2A,B**). Although the expression level of each protein was lower than that of Cor413im1-pA (**Figures 2A,B**), it was apparent that the full-length chimeric proteins were indeed expressed in *Arabidopsis*. Based on these data, we concluded that those four chimeric bicarbonate transporters were stably expressed in *Arabidopsis*.

### Localization of Chimeric Proteins Within Chloroplasts

**FIGURE 3:**
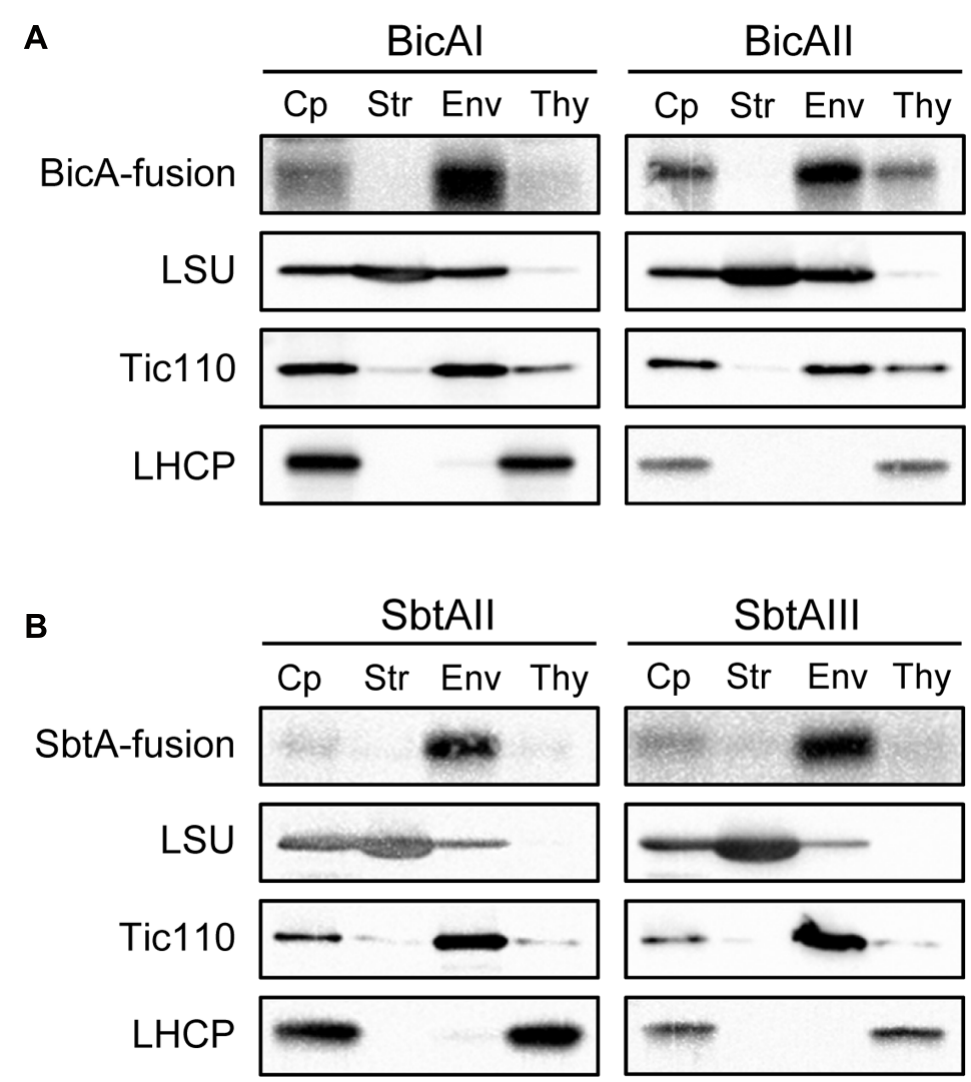
**Localization of chimeric BicA **(A)** and SbtA **(B)** in chloroplasts.** Isolated chloroplasts (Cp) were fractionated into stroma (Str), envelope (Env), and thylakoid (Thy) fractions. The protein ratio of Cp:Str:Env:Thy used in these analyses was consistently 3:3:1:1.5. Each fraction was resolved by SDS-PAGE and immunoblotted with antibodies against protein A (BicA- and SbtA-fusion), LSU, Tic110, or LHCP.

Next, we investigated the localization of these chimeric bicarbonate transporters within the chloroplasts. To this end, we isolated intact chloroplasts from these transgenic plants. Those chloroplasts were further fractionated into stroma, envelope, and thylakoid fractions (**Figures 3A,B**). The purity of each fraction was confirmed using marker proteins such as large subunit (LSU) of Rubisco (stroma), Tic110 (envelope), and light-harvesting complex protein (LHCP; thylakoid). As shown in **Figures 3A,B** (lanes Cp), all chimeric bicarbonate transporters were localized within the chloroplasts. Notably, each chimeric protein was found to be highly enriched in the envelope fraction (**Figures 3A,B**, lanes Env), indicating that the vast majority of these proteins are localized to the envelope membranes of chloroplasts. The level of enrichment of each chimeric protein in the envelope fraction was as high as Tic110, a genuine chloroplast IEM protein (Supplementary Figure S2). A previous study showed that the vast majority of BicA was targeted to the thylakoid membrane instead of the IEM when it was expressed from the plastid genome (Pengelly et al., 2014). We also observed that a small amount of each chimeric protein was fractionated into the thylakoid fraction (**Figures 3A,B**, lanes Thy). However, those are likely to be contaminants, as a small portion of Tic110 was also observed in the thylakoid fraction (Tic110 in **Figures 3A,B**, lanes Thy). In fact, the thylakoid marker protein, LHCP, was virtually undetectable in the envelope fraction (Supplementary Figure S2). Hence, we concluded that the vast majority of each chimeric bicarbonate transporter was specifically targeted to the envelope membranes of chloroplasts.

### Each Chimeric Bicarbonate Transporter Exists as an Inner Envelope Membrane Protein of Chloroplasts

**FIGURE 4:**
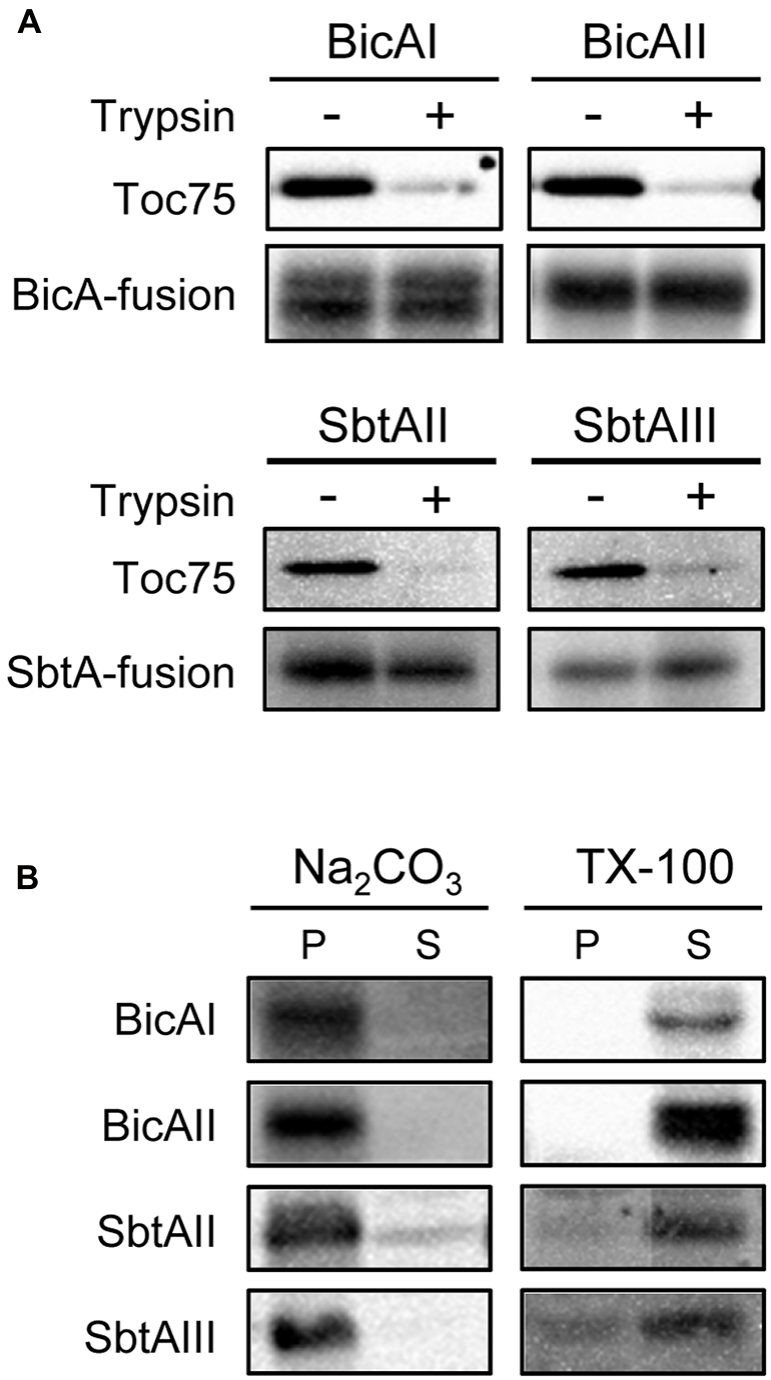
**Trypsin, alkaline, and detergent sensitivities of chimeric BicA and SbtA proteins in chloroplasts. (A)** Trypsin sensitivity of chimeric BicA and SbtA proteins in intact chloroplasts. Chloroplasts, equivalent to 25 μg chlorophyll, were treated with trypsin on ice for 30 min. The trypsin was inactivated and intact chloroplasts were re-isolated, resolved by SDS-PAGE, and immunoblotted with the antibody against protein A. The protease sensitivity of the outer envelope membrane protein, Toc75, was included as a positive control to confirm the validity of experiments. **(B)** Localization of chimeric BicA and SbtA proteins in the soluble and membrane fractions of chloroplasts. Chloroplasts were lysed in the buffer containing 0.2 M Na_2_CO_3_, pH 12 (Na_2_CO_3_), or 1% Triton X-100 (TX-100), and separated into insoluble (P) and soluble (S) fractions. The extracts were resolved by SDS-PAGE and immunoblotted with antibodies against protein A (BicA and SbtA).

The fact that each chimeric protein is enriched into the envelope fraction prompted us to further investigate the nature of these proteins in detail. We next investigated whether each chimeric transporter is an outer or inner envelope membrane protein. We isolated intact chloroplasts from transgenic plants expressing chimeric bicarbonate transporters and treated them with trypsin. Trypsin permeates the outer envelope membrane, but not the IEM, of intact chloroplasts (Jackson et al., 1998). As expected, the outer envelope membrane protein Toc75 was digested by trypsin (**Figure 4A**, Toc75). In contrast, all the chimeric bicarbonate transporters were resistant to the trypsin treatment (**Figure 4A**), indicating that those chimeric proteins are localized to the IEM.

Finally, we examined whether these chimeric proteins were integrated into the IEM, or peripherally associated with the IEM. When intact chloroplasts were solubilized with 1% Triton X-100, and fractionated into soluble and insoluble fractions, all chimeric proteins were partitioned into the soluble fraction (**Figure 4B**, TX-100). In contrast, all these proteins were resistant to alkaline extraction (**Figure 4B**, Na_2_CO_3_). These data indicate that each chimeric protein is an integral membrane protein at the chloroplast IEM.

Overall, our results indicate that the chimeric bicarbonate transporters fused to the IEM protein, Cor413im1, were specifically and efficiently targeted to the chloroplast IEM.

### Bicarbonate Transporters Can Reside in the Chloroplast IEM Even After the Removal of the IEM Targeting Signal

**FIGURE 5:**
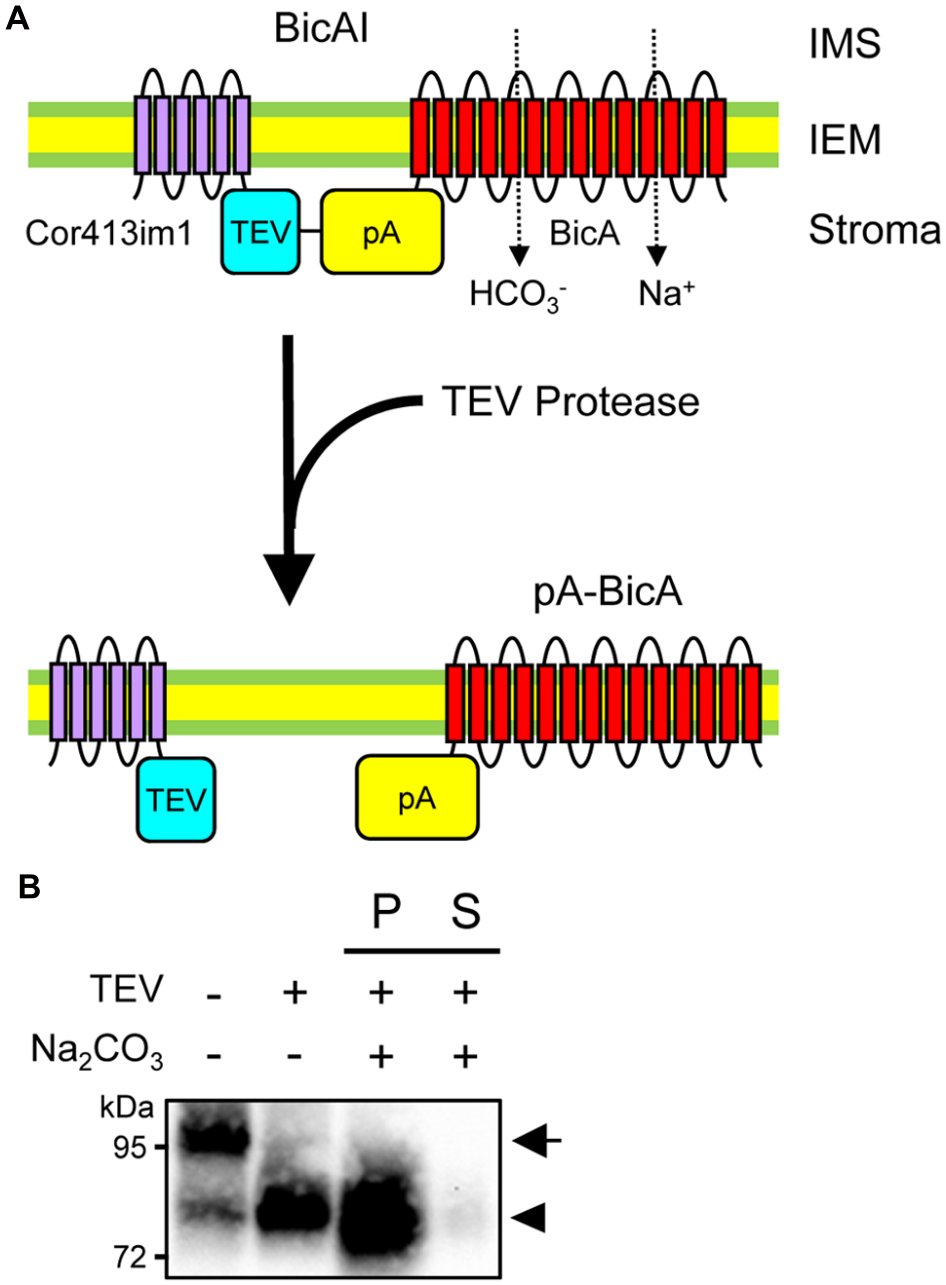
**Membrane integration of BicA portion in the absence of Cor413im1. (A)** A scheme to prove membrane integration of BicA in the chloroplast IEM. Topology of Cor413im1 and BicA portions of BicAI chimeric protein in the model is based on the published literature and protease sensitivity shown in **Figure 4A**. In the absence of the TEV protease, the TEV protease cleavage site (TEV), and the protein A portion (pA) of the BicAI chimeric protein, is assumed to localize in the stroma (upper). When the inside-out envelope membrane vesicles are treated with TEV protease, the Cor413im1 portion is cleaved from the chimeric protein, leading to the production of the new chimeric protein, pA-BicA. **(B)** Cleavage of BicAI by TEV protease and the resistance of pA-BicA to Na_2_CO_3_ treatment. Inside-out envelope membrane vesicles were treated with TEV protease. An aliquot was further lysed in the buffer containing 0.2 M Na_2_CO_3_, pH 12, and separated into insoluble (P) and soluble (S) fractions. The arrow indicates the position of BicAI. The position of pA-BicA is indicated by an arrowhead.

Although chimeric BicA and SbtA were integrated into the IEM, it is still unclear whether the bicarbonate transporters themselves are embedded to the IEM. For instance, one can argue that the Cor413im1 portion of each chimeric protein can serve as a membrane anchor, such that the chimeric proteins can reside in the IEM without the integration of the bicarbonate transporters into the IEM. To address this concern, we investigated if the removal of the Cor413im1 portion from BicAI affects the membrane localization of the BicA portion or not. As shown in **Figure 5A**, BicAI has a TEV protease cleavage site between Cor413im1 and protein A. Treatment of the inside-out envelope membrane vesicles of BicAI allows the removal of Cor413im1 from the chimeric protein, resulting in the creation of the pA-BicA chimeric protein (**Figure 5A**). Therefore, we investigated if the chimeric pA-BicA can reside in the IEM of chloroplasts or not. As predicted, the treatment of inside-out envelope membrane vesicles, isolated from BicAI chloroplasts, with TEV protease resulted in the production of a 75–80 kDa protein, which is pA-BicA (**Figure 5B**). This pA-BicA was resistant to alkaline extraction (**Figure 5B**). These data indicate that the BicA portion of the chimeric BicAI protein was integrated into the chloroplast IEM. Furthermore, the BicA portion can reside in the chloroplast IEM even after the removal of the IEM targeting signal.

## Discussion

Because of the growing demand on the food supply, increasing crop production by improving photosynthesis is becoming one of the major targets for plant scientists (Whitney et al., 2011; Price and Howitt, 2014a). One such approach is to install bicarbonate transporters into the chloroplast IEM (Price et al., 2011, 2013). However, due to the lack of knowledge concerning the targeting mechanism of chloroplast IEM proteins, it has been challenging to install bicarbonate transporters into the chloroplast IEM. In this study, we took advantage of the IEM targeting signal to specifically target bicarbonate transporters to the IEM. We established a method that allows the efficient and specific targeting of nuclear-encoded cyanobacterial bicarbonate transporters, BicA and SbtA, to the IEM of chloroplasts in *Arabidopsis*. Unlike plastome-expressed BicA, which are primarily targeted to thylakoid membranes (Pengelly et al., 2014), both chimeric bicarbonate transporters were almost exclusively targeted to the IEM within the chloroplasts (**Figures 3A,B**). Our results strongly suggest that the chloroplast IEM targeting signal, together with the transit peptide, can serve as a potential tool to install CCMs into the chloroplasts of land plants.

A previous study suggested that a certain class of transplastomic IEM proteins can be destined to the chloroplast IEM specifically. When the *TIC40* gene was expressed in the plastid genome, the plastome-expressed Tic40 protein was properly targeted, processed, and inserted into the IEM (Singh et al., 2008). Furthermore, chloroplasts in the transformed plants exhibited massive proliferation of the chloroplast IEM, and accumulated large amounts of plastome-expressed Tic40 (Singh et al., 2008). In contrast, when cyanobacterial BicA was transformed into the plastid genome, chloroplasts in transformed plants failed to accumulate the plastome-expressed BicA at the IEM (Pengelly et al., 2014). Instead, the majority of BicA was targeted to the thylakoid membranes. The reason why plastome-expressed BicA was targeted to the thylakoid membranes can be explained by the fact that a vast majority of IEM proteins are not inserted into the IEM from the stroma (Oh and Hwang, 2015). Among the nuclear-encoded IEM proteins, Tic40, and Tic110 have been demonstrated to be re-inserted into the IEM from the stroma, and utilize soluble intermediates (Lubeck et al., 1997; Li and Schnell, 2006; Tripp et al., 2007). In contrast, the vast majority of other proteins tested to date appear to be targeted to the IEM by a stop-transfer mechanism, and do not utilize soluble intermediates (Li and Schnell, 2006; Tripp et al., 2007; Firlej-Kwoka et al., 2008; Viana et al., 2010; Froehlich and Keegstra, 2011; Okawa et al., 2014). Furthermore, it appears that hydrophobic proteins lacking the IEM targeting signal seem to be mistargeted to the thylakoid membrane (Okawa et al., 2014). When truncated Cor413im1 lacking IEM targeting signal was expressed in *Arabidopsis*, the majority of Cor413im1 was destined to the thylakoid membrane (Okawa et al., 2014). Hence, we speculate that, without IEM targeting signal, it will be challenging to install plastome-expressed bicarbonate transporters into the chloroplast IEM specifically.

Intriguingly, some of the chimeric proteins were undetectable in transgenic *Arabidopsis*. We speculate that the transmembrane topology of each chimeric protein may be attributable to those observations. According to a topology prediction in the previous study, BicA and SbtA possess 14 and 10 transmembrane segments (Price, 2011; Price and Howitt, 2014b). Both the N- and C-termini of BicA are predicted to face the cytoplasm in cyanobacteria (Price and Howitt, 2014b). In contrast, both the N- and C-termini of SbtA seem to localize in the periplasm (Du et al., 2014). Because the Cor413im1 protein has 6 transmembrane segments and both N- and C-termini faces the stroma (Okawa et al., 2008) (**Figure 1B**), we assume that BicAI and BicAII are likely to possess desirable transmembrane topology for the transport of bicarbonate into chloroplasts. Likewise, the N-terminus of the K124 portion of the SbtAIII construct is likely to be exposed to the intermembrane space because K124 is predicted to have five transmembrane segments (**Figure 1B**). Hence, it is conceivable to speculate that SbtAIII likely exhibits a desirable transmembrane topology for the transport of bicarbonate into chloroplasts. Overall, the chimeric proteins expressed in our study seem to possess the desirable transmembrane topology, with the exception of SbtAII.

**FIGURE 6:**
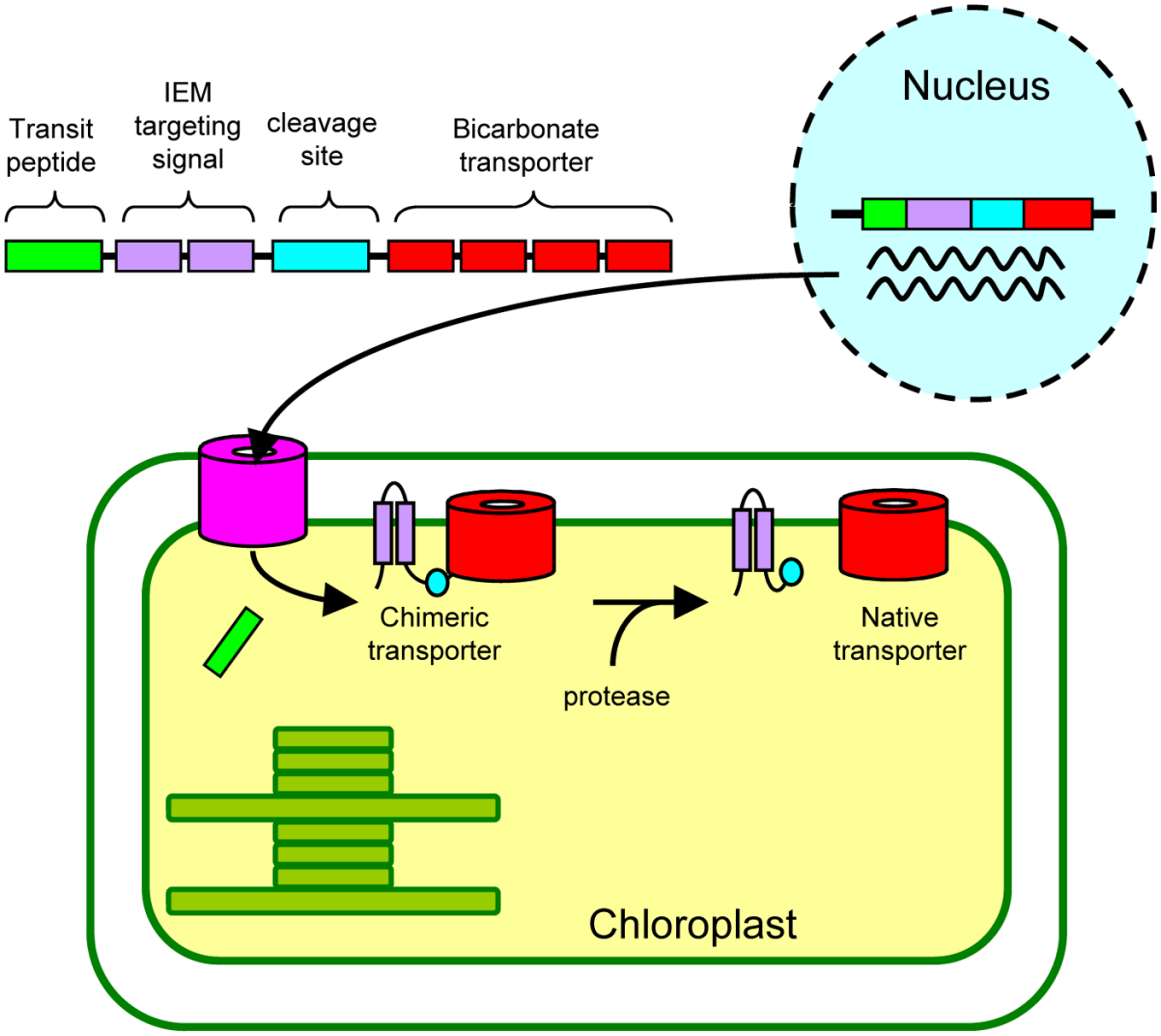
**A proposed approach to install cyanobacterial bicarbonate transporters, encoded by the nuclear genome, into the inner envelope membranes (IEM) of chloroplasts in land plants**.

Finally, we propose a possible approach by which we can install active cyanobacterial bicarbonate transporters into the chloroplast IEM (**Figure 6**). As demonstrated in our current study, the addition of the IEM targeting signal to the bicarbonate transporters is necessary. Although we have used the full length and truncated Cor413im1, the IEM targeting signals can be further optimized for practical application (e.g., minimizing the length of an IEM targeting signal). These chimeric genes can be transformed into the nucleus. Unlike chloroplast transformation, nuclear transformation can be performed in numerous plant species. Once the chimeric proteins are targeted to the chloroplast IEM, they must be activated, allowing the active incorporation of bicarbonate into chloroplasts. Activity of the chimeric transporter may be sufficient to transport bicarbonate into chloroplasts, but it is entirely possible that addition of an IEM targeting signal could inhibit the activity of the bicarbonate transporter. If this is the case, the IEM targeting signal can be removed from the chimeric protein using a protease, resulting in the production of the “native” bicarbonate transporter at the IEM. Recent studies have shown that the β-carboxysome-like structure can be reconstituted within chloroplasts (Lin et al., 2014a,b). Hence, once the activities of the bicarbonate transporters at the chloroplast IEM are evaluated, simultaneous installation of carboxysomes, as well as bicarbonate transporters, would be possible to improve photosynthesis in C_3_ plants.

## Conclusion

We successfully installed chimeric cyanobacterial bicarbonate transporters into the chloroplast IEM. Although the effects of those chimeric bicarbonate transporters on photosynthesis remain to be characterized, the specific and efficient targeting of cyanobacterial bicarbonate transporters to the chloroplast IEM serve as a milestone toward achieving “turbocharged photosynthesis.”

## Author Contributions

TI and YI-I designed and supervised the research. SU, FA, and TI performed research. SU, YI-I, and TI analyzed data. SU and TI wrote the paper.

## Conflict of Interest Statement

The authors declare that the research was conducted in the absence of any commercial or financial relationships that could be construed as a potential conflict of interest.
