# Supplementary material for: Specific and Efficient Targeting of Cyanobacterial Bicarbonate Transporters to the Inner Envelope Membrane of Chloroplasts in Arabidopsis
Source: Front Plant Sci. 2016 Feb 2;7:16. doi: 10.3389/fpls.2016.00016 (PMC4735556; doi:10.3389/fpls.2016.00016)
Supplement: Supplementary file 1 [file Presentation_1.PDF]

Supplementary Figure S1

A

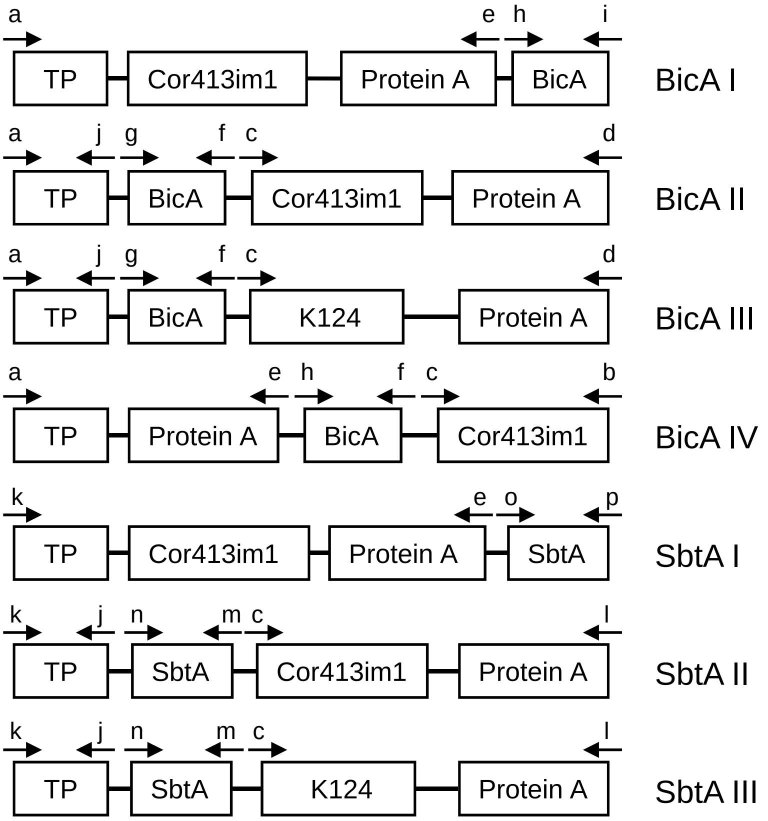

B

a, 5’- CAGAAGAAAGCCATGGCGAGTCTCTGTCTCTCATCGT-3’
b, 5’- TGTTGATTCATCTAGATTATAGCAACAAAAGGAGCGATAC-3’
c, 5’-GGAGGCAGTGGTGGATCAGCTGCTCCGATTTCTGCCAAT-3’
d, 5’-TGTTGATTCATCTAGAATCATTTTGGTGCTTGAGCAT-3’
e, 5’-GTCGAGAGGGATGTCGTAGTCTTTTGGTGCTTGAGCAT-3’
f, 5’-TCCACCACTGCCTCCGTATGTGGTCTGGACGGAAGAACT-3’
g, 5’-CTGTACTTCCAGGGAATGCAAATAACTAACAAAATTCAT-3’
h, 5’-GACATCCCTCTCGACATGCAAATAACTAACAAAATTCAT-3’
i, 5’-TGTTGATTCATCTAGAATCAGTATGTGGTCTGGACGGAAGA-3’
j, 5’-TCCCTGGAAGTACAGGTTCTC-3’
k, 5’-GGACTCTTGACCATGGCGAGTCTCTGTCTCTCATCG-3’
l, 5’-GGTGGTGGTGGCTAGCTCATTTTGGTGCTTGAGCAT-3’
m, 5’-TCCACCACTGCCTCCACCTGCACCAAGGGTCTGGGCAAG-3’
n, 5’-CTGTACTTCCAGGGAATGGATTTTTTGTCCAATTTCTTGACGG-3’
o, 5’-GACATCCCTCTCGACATGGATTTTTTGTCCAATTTCTTGACGG-3’
p, 5’-GGTGGTGGTGGCTAGCTTAACCTGCACCAAGGGTCTGGGCAAG-3’

Supplementary Figure S1. List and combination of primers used to amplify each portion of chimeric BicA and SbtA constructs.
A. Schematic diagram of chimeric constructs used in this study. PCR primers used for amplification of each fragment are indicated by black arrows.
B. Nucleotide sequence of the primers indicated in A.

Supplementary Figure S2

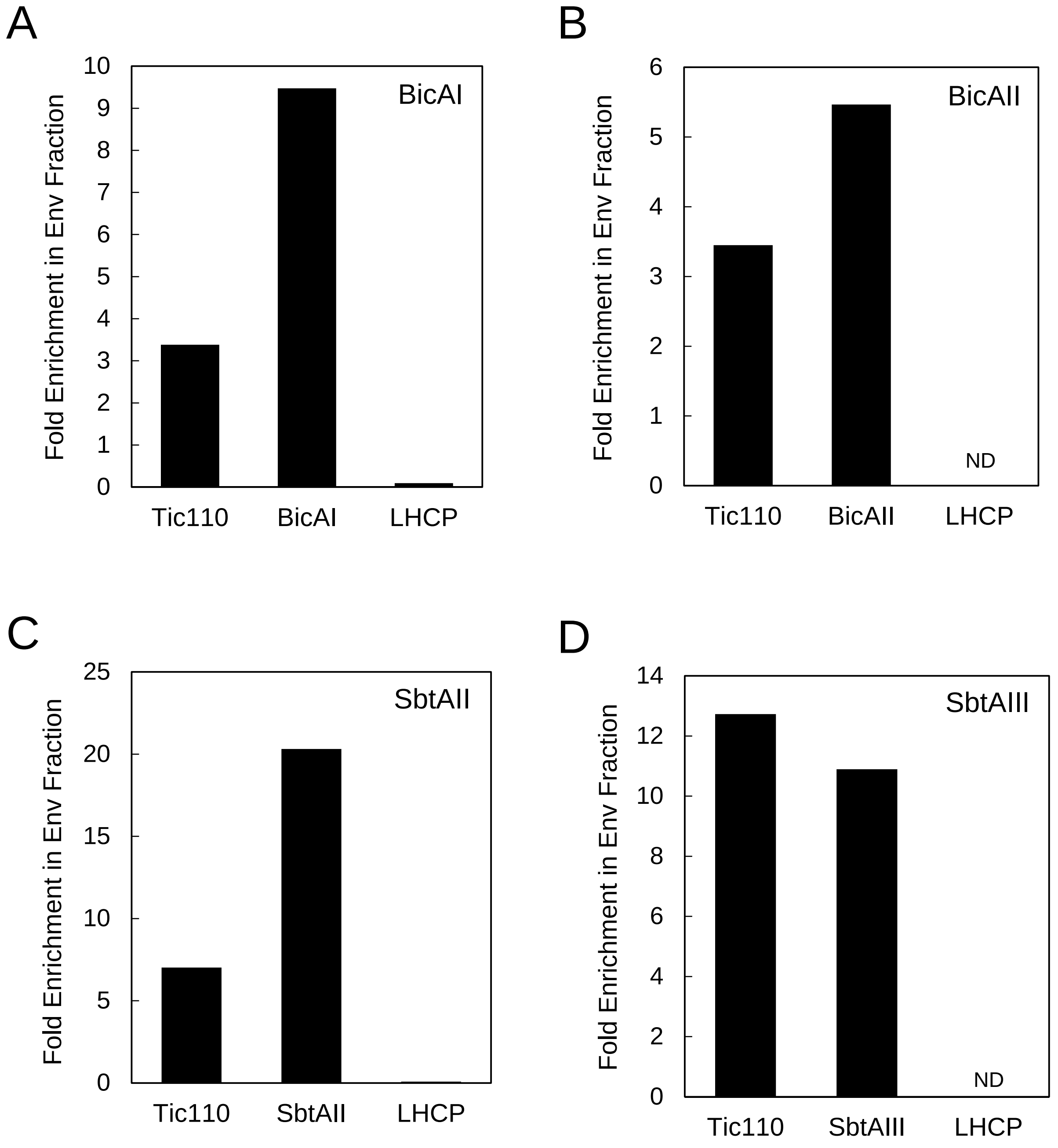

Supplementary Figure S2. Fold enrichment of each chimeric protein in the envelope fraction.

The signal intensity of each band (lanes Cp and Env in Figure 3) was first measured by densitometry and the signal intensity per microgram protein was calculated based on the amount of protein loaded. Then, the value for the envelope fraction was divided by the value for the total chloroplast fraction to estimate the fold enrichment in the envelope fraction compared to the total chloroplast. Fold enrichment of BicAI (A), BicAII (B), SbtAII (C) and SbtAIII (D) in the envelope fraction is shown in each panel. As controls, fold enrichment of Tic110 and LHCP in the envelope fraction was also calculated. ND, not detected.
